# Anthropogenic Effects on Natural Mammalian Populations: Correlation Between Telomere Length and Coal Exposure

**DOI:** 10.1038/s41598-019-42804-8

**Published:** 2019-04-19

**Authors:** Cristina A. Matzenbacher, Juliana Da Silva, Ana Leticia H. Garcia, Mónica Cappetta, Thales R. O. de Freitas

**Affiliations:** 1 Department of Genetics, Federal University of Rio Grande do Sul, C.P. 15053, 91501-970 Porto Alegre, RS Brazil; 2 Laboratory of Genetic Toxicology, Lutheran University of Brazil, ULBRA, Canoas, 92425-900 Rio Grande do Sul Brazil; 3 Laboratory of Ecotoxicology, Postgraduate Program in Environmental Quality, University Feevale, ERS-239, 2755, 93525-075 Novo Hamburgo, RS Brazil; 4 Laboratorio de Epidemiología Genética, Departamento de Genética, Facultad de Medicina, Universidad de la República, Montevideo, Uruguay

**Keywords:** Conservation biology, DNA methylation, Environmental impact

## Abstract

The Candiota coal mine in Rio Grande do Sul (RS) is one of the largest in Brazil. Coal is a fossil fuel that causes environmental impacts from its extraction to combustion due to the release of different agents, such as polycyclic aromatic hydrocarbons (PAH) and heavy metals. C*tenomys torquatus* are herbivorous and subterranean rodents that dig tunnels with their paws and teeth and can be exposed to coal through contaminated food. Exposure to pollutants can cause DNA damage and affect different tissues, inducing alterations in the population structure and genetic diversity. Our study aimed to evaluate the effect of exposure to coal and its derivatives on the *C. torquatus* population and to examine the relationship of coal exposure with variations in absolute telomere length (aTL), global DNA methylation and genotoxicity. Our study showed an inverse correlation between telomere length and coal exposure in addition to an increase in DNA damage. The results indicate that coal and its byproducts can contribute to the alteration of the *C. torquatus* population structure, as evidenced by a reduction in the number of adults.

## Introduction

The purpose of ecotoxicological studies is to detect a cause-effect relationship between different ecosystems, from the molecular level to the entire ecosystem, and complex chemical mixtures of pollutants^1^. Many of the contaminants in environment affect organisms directly, which present acute effect and fast detection, and can cause physiological disorders, such as endocrine disruption, abnormal development and/or decreasing the lifetime, reducing reproductive parameters and altering the sex ratio^2,3^. In large populations, mutations can be eliminated by selection; however, in small populations, selection is less effective, and genetic drift is one of the factors that can eliminate a mutation^4^ that is more common in small populations than selection and gene flow^5^. The continuous progression and decline of “fitness” in small populations due to the fixation of characteristics are not changes that occur without consequences^6^. Since the mutations induced by pollutants generally do not occur in a single individual in the exposed population, the detection of changes in the genome plays a fundamental role in species conservation. These exposure-induced mutation can lead to incorrect gene expression and/or damage to somatic cells, which can reduce an individual’s health and reproductive capacity, disrupt development, alter the sex ratio by reducing the effective population size, decrease the genetic diversity, and lead to the end of the viability of the population^7^.

Coal is a fossil fuel that causes impacts through environmental pollution from its extraction to its combustion. A wide range of coal dust and coal byproducts contribute to the pollution of the atmosphere^8^, water and soil^9,10^. Inorganic elements (metals) and polycyclic aromatic hydrocarbons (PAH), which are found in coal, can induce DNA damage by introducing a cellular oxidative imbalance by the generation of several reactive oxygen species (ROS)^11–13^. Telomere, a noncoding DNA sequence at the end of eukaryotic chromosomes, has a sequence of adjacent triple-guanine nucleotides (TTAGGG) that makes them highly sensitive to oxidative stress and single-strand breaks^14^. It is known that DNA methylation has a function in telomere length variability^15,16^. DNA methylation patterns are formed by a family of DNA methyltransferases enzymes that transfer a methyl group from s-adenosyl-1-methionine (SAM) to the 5-position of cytosine forming 5-methyl-2′-deoxycytidine (5-mdC), primarily found in CpG dinucleotides^17^. Previous studies^18,19^ showed an inverse correlation between PAH and telomere length in different types of human cells and tissues. Stauffer *et al*.^14^ studied the association among exposure to heavy metal pollution, oxidative stress and telomere damage in two passerines, the great tit (*Parus major*) and the pied flycatcher (*Ficedula hypoleuca*). Recently, studies concerning the effects of environmental pollution on DNA methylation in human and animal, found an association between levels of DNA methylation and exposure to hydrocarbons and inorganic elements^17,20^. An *in vitro* study suggested a relationship between ROS and global DNA methylation^18^.

The Presidente Médici thermoelectric plant is a complex composed of three plants: Candiota I, inaugurated in 1961; Candiota II, which has been operating since 1974; and Candiota III, which entered into commercial operation in 2011. All the coal used in this plant comes from the Candiota coalfield, one of the largest coal reserves in Brazil, which is located in the municipality of Candiota, RS, 400 kilometers from Porto Alegre (State Capital)^21^. Several studies have been conducted characterizing coal and its byproducts on power plants in Candiota (RS, Brazil) in addition to evaluating their effects on different organisms. Including a study performed by Siva *et al*.^22–24^, who observed an increase in DNA damage in *C. torquatus*.

**Figure 1:**
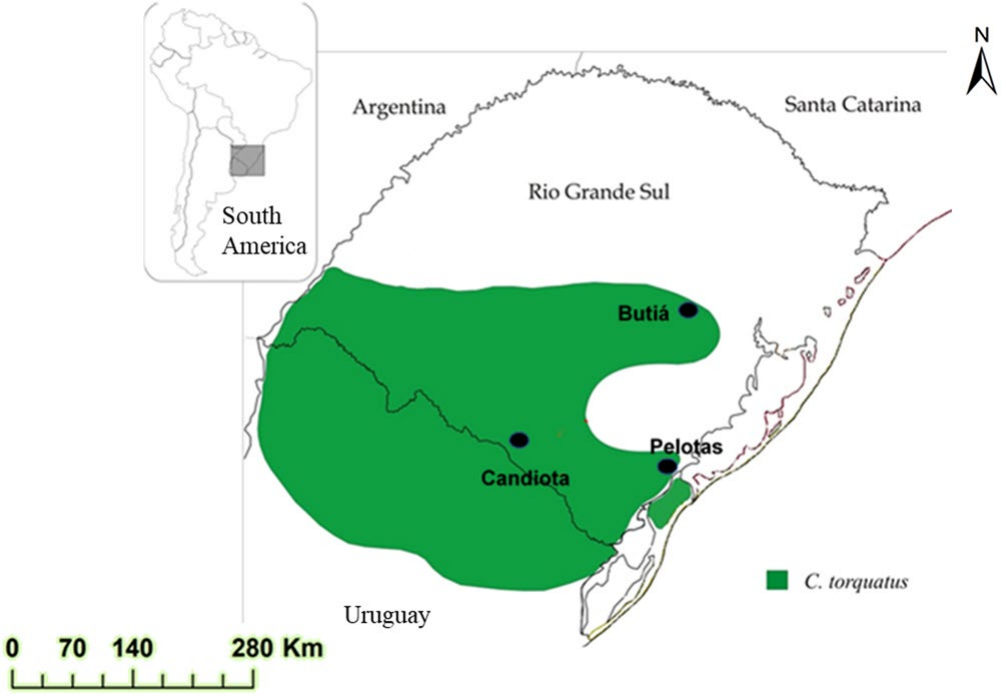
Geographic distribution of *C. torquatus*, which coincides with the geographic distribution of coal reserves and power plant Candiota (modified from Fernandes *et al*.^62^).

The collared tuco-tuco, *C. torquatus* Lichtenstein 1830, is a subterranean rodent with a diploid number (2n) of 40, 42, 44 and 46 chromosomes and a fundamental number (FN) of 72 (Refs.^25,26^). This species is present in the fields of the Pampas ecoregion, from the central area of the RS, in southern Brazil, to the central-north region of Uruguay^27^. The geographical distribution of *C. torquatus* in the RS almost coincides with the geographic distribution of the state’s coal reserves^28^ (Fig. 1). The presence of PAH was found in its coal, bottom and fly ash samples^29^, metals were detected in soil samples^30^ and Zn, Ni, Cu, Cd, V and Pb were detected in the tissues of the subterranean rodent *C. torquatus*^23^. In addition, studies with organisms have shown DNA damage in plants (*Baccharis trimera*)^9,31^, snail (*Helix aspersa*)^10^ and coal workers’ blood^30,32,33^. To our knowledge, no previous studies have examined the effects of coal and its byproducts on telomere length or DNA global methylation in tuco-tuco.

In this study, we aimed to evaluate the effect of coal exposure and its derivatives on the *C. torquatus* population using absolute telomere length and global DNA methylation measurements, as well as correlate these results with DNA damage obtained previously by Silva *et al*.^19–21^. We hypothesized that the animals living near coal reserves and coal exploration regions that previously reflected in high levels of DNA damage^22^ would show high levels of genomic instability now measured by molecular techniques.

## Results

### Population Size Monitoring

To monitor the population size, we used 198 rodents trapped over two years by Silva *et al*.^22^ in three regions: (i) Pelotas, with 77 animals captured and 18% recaptured; (ii) Butiá, with 61 captures and 20% recaptured; (iii) Candiota with 60 animals captured but only 10% recaptured. The female: male ratio was 2:1 in the Pelotas field, 3:1 in the Butiá field and 4:1 in the Candiota field. When comparing the regions with coal derivative exposure and without coal exposure, we verified that among the three age classes, the results were different (Table 1).

**Table 1:** Proportion of individuals by age from region with and without coal and derivatives exposition and population size estimative (Ne).

Age	Pelotas (%)	Butiá (%)	Candiota (%)
Juveniles	10	11	5
Subadults	34	35	49
Adults	53	54	46
Ne ( ± SD)^a^	22 (7)	17 (11)	37 (16)*

^a^By Lincoln-Petersen method; *Significant in relation to Pelotas and Butiá at P < 0.05, ANOVA.

### Comet Assay

**Figure 2:**
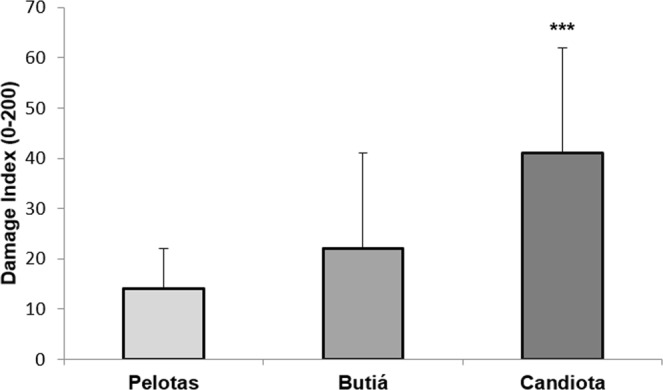
Detection of DNA damage (mean ± SD) in the blood leukocytes of *C. torquatus* from Pelotas (N = 30), Butiá (N = 14), and Candiota (N = 22). ***Significant compared to Pelotas at P < 0.001, ANOVA (Dunnett).

**Figure 3:**
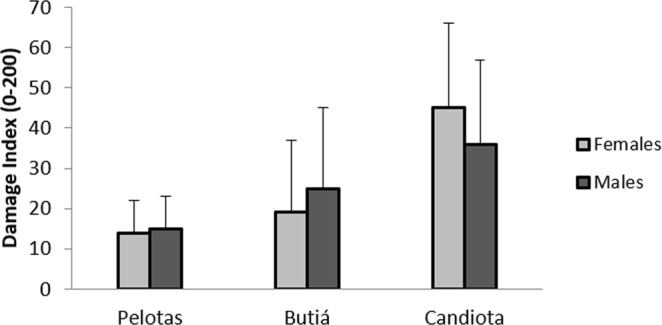
Mean values (mean ± SD) of the damage index in the leukocyte cells of *C. torquatus* by site and sex. Pelotas (female = 22; male = 8), Butiá (female = 11; male = 3) and Candiota (female = 19; male = 3). No significant differences were found.

**Figure 4:**
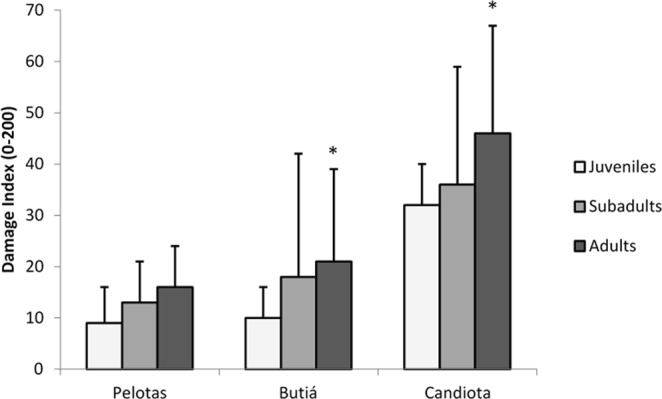
Comparisons between age, site, and damage index - DI (mean ± SD) of blood samples from *C. torquatus*. *Significant compared to juveniles from the same site at P < 0.05; ANOVA (Dunnet). Pelotas (5 juveniles, 8 subadults and 17 adults), Butiá (1 juvenile, 4 subadults and 9 adults) and Candiota (1 juvenile, 18 subadults and 3 adults).

Regarding the Comet assay (CA) results (data obtained by Silva *et al*.^20^), 30 rodents were analyzed as the control group (Pelotas), and 36 rodents were analyzed as the exposure group (14 Butiá and 22 Candiota), considered here are only the individuals utilized for this study. Figure 2 shows a significant increase in DI (damage index, details see Material and Methods) in the Candiota rodents compared with the Pelotas rodents. Males and females from Candiota demonstrated a DI significantly higher than males and females from the control location, Pelotas, but no difference between the sexes of each group was observed (Fig. 3). Regarding age, Fig. 4 shows that juveniles have a lower DI than subadults and adults at each site. Adults from Butiá and Candiota displayed a DI significantly different from the juveniles of the same site (P < 0.05).

### Telomere Length

**Figure 5:**
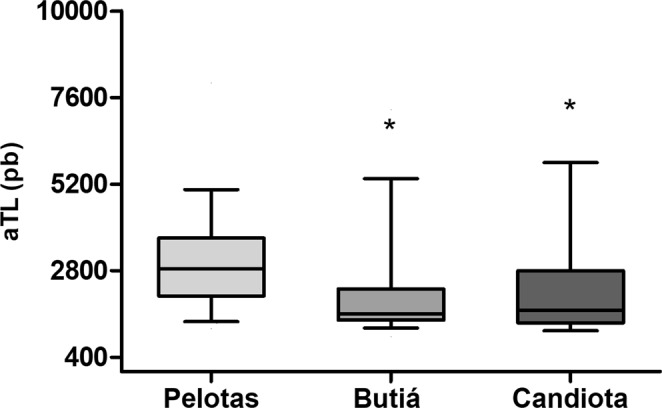
Telomere length of nonexposed (Pelotas) and exposed (Butiá and Candiota) *C. torquatus* individuals. *Significantly different from the nonexposed group at P = 0.01; ANOVA (Kruskal Wallis).

To evaluate absolute telomere length (aTL), 30 rodents were used as the control group (22 females and 8 males), and 36 rodents were used as the exposure group (30 females and 6 males). There is an inverse relationship between the absolute telomere length and the exposure to coal (P = 0.01): the control group had a higher aTL than the exposure group (Fig. 5). Moreover, we dismissed sex (r = 0.042; P = 0.731) and age (r = 0.049; P = 0.706) as confounding factors in the correlation between aTL and coal effects. Additionally, we did not find significant differences in the telomere length of the different tissue samples evaluated.

### Global DNA Methylation

**Figure 6:**
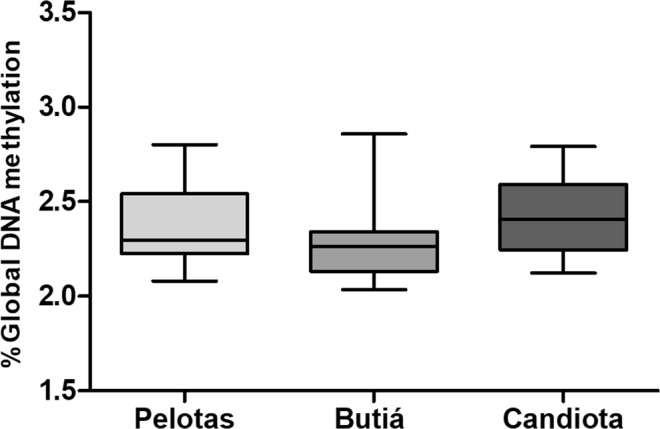
Proportion Global DNA methylation of nonexposed (Pelotas) and exposed (Butiá and Candiota) *C. torquatus* individuals.

We measured global DNA methylation levels in tissues from tuco-tuco in Pelotas, including 30 (22 females and 8 males) individuals as the control group, and in Butiá or Candiota, including 35 (30 females and 5 males) as the coal-exposed individuals, by comparing the relative levels of 5-mdC. We did not find a significant difference in the global DNA methylation between the cities. The average (±SD) methylation levels were 2.3% (±0.19) in Pelotas, 2.27% (±0.18) in Butiá and 2.41% (±0.2) in Candiota (Fig. 6). We also dismissed sex (r = 0.095; P = 0.456) and age (r = 0.006; P = 0.965) as confounding factors in the correlation between the levels of 5-mdC and coal effects, and we did not find significant differences in the levels of 5-mdC of the different tissue samples evaluated.

## Discussion

Previous^22,23^ and current studies indicate that coal and its byproducts induce DNA damage in tuco-tucos with no a difference between males and females. Silva *et al*.^23^ demonstrated an association between DNA damage and hydrocarbons level in soil. This finding coincides with Zitko *et al*.^34^, who measured PHA in harp seal beaters (*Phoca groenlandica*) and did not find a difference between males and females. The main cellular mechanism of DNA damage discussed by the authors was the oxidative lesions caused by PHA^9,18,35^.

Telomeres are known to be highly susceptible to oxidative damage and telomere attrition can be accelerated by different environmental factors, such as coal^32^. The ability of ROS to induce 8-oxodG within the GGG triplet found in the telomeric sequence (TTAGGG) is why telomeres are particularly susceptible to oxidative stress-induced damage and chromosome instability, as explained in Coluzzi *et al*.^36^. Our results revealed a significant reduction in aTL in the rodents exposed to coal (Candiota and Butiá) compared with the aTL of the nonexposed rodents (Pelotas). Many variables can influence the loss of telomere length, such as age^37^, sex^38^, and tissue^39^. Thus, we performed a correlation analysis in the evaluated population to test whether some of those factors could be influencing the reduction in telomere length; however, our results did not support this correlation. We observed that exposure to coal was significantly associated with a decrease in aTL even after adjusting for these variables. Daniali *et al*.^39^, in a study examining telomere shortening in human somatic tissues, found that in the tissues analyzed (leukocytes, muscle, skin and fat), there were similar rates of age-dependent aTL attrition in adulthood, and the aTL among tissues did not show significant changes with age. This variation in aTL across somatic tissues is also observed in other mammals^40^. Kirby *et al*.^41^ studied the relationship between environmental variables, such as habitat productivity, human development, elevation, and latitude, and individual characteristics, like age, sex, body size and genetic kinship, in *Ursus americanus* and did not find a relationship between telomere length and individual characteristics. Another study found a relationship between shortened telomeres in the blood and exposure to persistent organic pollutants (POPs) using *Rissa tridactyla*, an arctic seabird species, and the most extensive shortening was observed in females^38^. We did not find a relationship between aTL and sex, despite the trend of more telomere shortening (not significant) in male tuco-tucos than in female tuco-tucos in a region exposed to coal. These results indicate that coal and coal byproducts may contribute to telomere shortening in the *C. torquatus* population, and these results can also affect other mammals in the region.

DNA global methylation is an epigenetic alteration that plays a role in regulating cellular processes, including genomic instability and gene expression^42^. Many human and animal studies have investigated the effects of environmental pollution and have reported an association between DNA methylation levels and environmental exposures, including lead, arsenic, benzene, Cr(VI), POPs and air pollutants^17,20,43^. Additionally, the methylation of the sub-telomeric region is correlated with aTL and can be an important region for the epigenetic regulation of telomere length^16,44^. In this study, we did not find a relationship between global DNA methylation and coal exposure, and no correlation was found between aTL and DNA methylation. De Souza *et al*.^32^ observed DNA instability and an inverse correlation between telomere length and coal exposure in the mine workers but did not detect correlations between aTL and DNA methylation. We also performed correlations examining the relationship of the percentage of global DNA methylation with sex, age and tissues; however, no correlation was found, probably due to our small sample size, despite detecting more global DNA methylation, with no significant difference, in exposed males than in exposed females. The effect of sex, age and tissue variations in the levels of DNA methylation has been widely discussed, as different studies report different findings, especially when considering the possibilities of inter-individual differences^45–48^. These variations can occur due to the differences in the methods used to quantify DNA methylation, the heterogeneity of tissue types, the sample size and the type of analysis used to evaluate the global methylation content or the locus specific methylation.

Coal extraction and burning have caused environmental and human health problems in South Brazil, in both mining regions and nearby regions (e.g., acid rain in Uruguay)^49,50^. However, analytical techniques that characterize the levels of known contaminants do not provide insight into the biological risks associated with pollution^51^ nor the effect on natural population structure. Although *C. torquatus* are subterranean rodents, they normally come up to the surface to collected food and clean their burrows. They are herbivorous animals, digging their den with their paws and teeth. Some animals that are bred in contaminated environments are directly exposed to these contaminants, but herbivores, such as the subterranean rodents analyzed in this study, can be exposed through contaminated food, as observed by Menezes *et al*.^31^ in plants from Candiota, which had hight level of inorganic elements. Thus, these animals expose themselves to the contaminants in the soil and plants, in their microenvironment and in the atmosphere in regions with or without pollutants.

Our results demonstrated a possible relationship between age and DNA damage. Adults and juveniles of tuco-tucos from Candiota had higher DNA damage than rodents of the same age group from Butiá (Fig. 4). Increase genetic alterations, such as chromosomal structural aberrations and aneuploidies, are associated with aging^52^. Increase as these, in the levels of cell damage associated with age, were found in whales exposed to PHAs, with the adults showing significantly more cytochrome P450 expression than juveniles^53^. The data reveal that the estimated size of the population of Candiota (the region where the rodents had higher DNA damage) is larger than the population sizes at Pelotas and Butiá but there was lower frequency of recaptures in Candiota (Table 1). However, it can also be noted that the genotoxic damage was the greatest in the adult organisms, of which there were fewer in Candiota, leading to the assumption that - among others - there is an increase in mortality rate as a result of the probable cumulative damage. Additionally, Roratto *et al*.^54^ examined the phylogeographic pattern of *C. torquatus* and found approximately 40 haplotypes. In the regions where the populations were analyzed that were under the influence of coal, four haplotypes were found in Minas do Leão, a region that only extracts coal and is also at the center of the distribution range. In Pelotas, the location that served as control over the other two populations, there were two haplotypes found in a peripheral geographical region. Candiota, which is the center of our toxicology analysis, showed only one haplotype and is also located centrally in the geographical distribution of the species. These data indicate low genetic variability in Candiota. Regarding genetic variability, the same relationship was observed as with DNA damage; however, in the ascending order was reversed, i.e., Candiota < Pelotas < Butiá. In a review by Hamilton *et al*.^7^, the disappearance of 16 fish species in Belews Lake in the USA was reported, which was assigned to selenium contamination from a coal ash disposal basin.

The results of our study describing the population size in the three evaluated cities suggest that coal exposure does not interfere with *C. torquatus* population size or sex ratio, but instead impacts the structure of the population, as reflected by adults representing the largest number of recaptured individuals. Furthermore, *C. torquatus* suffer DNA damage/instability, as observed in the comet assay (DNA damage), and telomere shortening, likely as a consequence of the oxidative damage that results from their exposure to a composite mixture, including inorganic elements such as those found in the soil^23,33^ and lung of *C. torquatus* and organic elements such as hydrocarbons^23^. The Candiota population is impacted by coal, and certainly other mammal populations could also be affected. Almeida and Freitas^55^ wrote about the effects of coal on the cranial morphology of *C. torquatus*. This study reports a relationship of environmental influences and pressures with ontogenetic trajectory. These results indicate that the vertebrate fauna should be constantly monitored, and here we suggest that species with a low “home-range” such as *Ctenomys* can be affected.

In conservation genetics, these analyses that were conducted with *C. torquatus* represent a new way to evaluate the anthropogenic effect on natural mammalian populations. We analyze three new points of view (genotoxicity, telomere length and methylation) as well as addressing conservation genetics, which is a field that is always concerned about the genetic diversity of small populations. Thus, these results, which showed a correlation of coal exposure with damage to the genome and decreased telomere length, suggest that such effects underlie the effects that have always been analyzed in conservation genetics, including are population parameters and genetic variability. Our study is important due to the emphasis on the viability of genotoxic assays and epigenetic tools in conservation studies. Currently, we are observing an increasing trend of cooperation among ecotoxicologists and conservationists, as evidenced by the growing number of studies with this approach, which can improve both conservation and evolution studies. More studies are needed to estimate the population size and increase the sample sizes to know the current status of these populations.

## Materials and Methods

### Approval and Accordance

#### Approval

This study was performed with the permission of the official Brazilian Environmental Protection Agency - IBAMA (14690-1).

#### Accordance

The sampling methodology used in this study was in accordance with all regulations and guidelines from the Ethics Committee on the Use of Animals of the Universidade Federal do Rio Grande do Sul (CEUA - 31925).

### Sampling

The samples used in this study were collected by Silva *et al*.^22–24^. *Ctenomys torquatus* (Ctenomyidae-Rodentia) individuals were captured at two locations in different environments in RS: the individuals in the control group were captured in Pelotas (31°S, 52°W), a region without a coal mine and power plant; and the individuals in the exposed group were captured at two locations, Butiá (30°S, 51°W), a region approximately 5 km from a strip coal mine, and Candiota (31°S, 54°W), a region about 2 km from the Presidente Médici coal power plant. All populations presented the same diploid number, 2n = 44 (Ref.^25^) (Fig. 1).

The natural populations of the rodents were monitored by Silva *et al*.^22–24^. The *C. torquatus* age groups were determined according to the^56^ method, which is based on body weight. Three age classes for *C. torquatus* were determined by weight: juveniles, females up to 125 g and males up to 135 g; subadults, females from 125 to 185 g and males from 135 to 225 g; and adults, above the limits of the subadults^22,23^.

The tissue samples used in this study to determined absolute telomere length (aTL) and global DNA methylation were obtained from the collection of the Laboratório de Citogenética e Evolução of the Universidade Federal do Rio Grande do Sul, and we used the samples that were available.

### DNA Extraction

DNA extraction was performed on the tissues from 30 controls (24 females and 6 males) and 36 exposed individuals (30 females and 6 males). Total DNA was extracted from liver, kidney, muscle and skin tissue samples using a standard phenol: chloroform protocol^57^.

### Genotoxicity Measurement by Comet Assay

The alkaline comet assay (CA) in blood cells was performed as described by Singh and Stephens^58^ as modified in Silva *et al*.^24^ for field work. Images of 25 randomly selected cells (in duplicate) were analyzed from each animal. Cells were visually classified according to tail size into five classes: undamaged (0) to maximally damaged (4), resulting in a single DNA damage score for each animal and consequently to each studied group. Therefore, the group’s damage index (DI) could range from 0 (completely undamaged, 50 cells X 0) to 200 (maximum damage, 50 cells X 4).

### Telomere Length Analysis by qPCR

Telomere length was measured from the total genomic *C. torquatus* DNA of 30 controls (22 females and 8 males) and 36 exposed individuals (30 females and 6 males) by using a real-time quantitative Polymerase Chain Reaction (qPCR) method following the protocol described by Callicott and Womack^59^, with slight modifications. The forward and reverse primer sequences for the telomeric region gene were 5′ CGG TTT GTT TGG GTT TGG GTT TGG GTT TGG TTT GGG TT 3′ and 5′ GGC TTG CCT TAC CCT TAC CCT TAC CCT TAC CCT TAC CCT 3′, respectively. Primers for the mouse 36B4 gene corresponded to the acidic ribosomal phosphoprotein PO (36B4) gene, which is well conserved. The forward and reverse primers used in the 36B4 portion of the assay were 5′ ACT GGT CTA GGA CCC GAG AAG 3′ and 5′ TCA ATG GTG CCT CTG GAG ATT 3′, respectively^59^. Each reaction examining the telomere and 36B4 fragments included 12.5 μl SYBR Green PCR Master Mix (Quatro G), 300 nM telomere primers (forward and reverse), 300 nM 36B4 forward primer and 500 nM 36B4 reverse primer, 20 ng genomic DNA, and enough water was added to yield a 25-μl reaction. Three 20-ng samples of each DNA mixture were placed in adjacent wells of a 96-well plate for the telomere and 36B4 assays and analyzed using the Step One Plus TM Real Time PCR System (Applied Biosystems, Foster City, CA, USA). For the telomere amplicons, qPCR was performed using the following reaction conditions: set at 95 °C for 10 min; followed by 30 cycles of denaturation at 95 °C for 15 s, and annealing and extension at 56 °C for 1 min. For the 36B4 amplicons, the reaction conditions were an initial step at 95 °C for 10 min followed by 35 cycles of data collection at 95 °C for 15 s, with 52 °C annealing for 20 s, followed by extension at 72 °C for 30 s.

Serially diluted DNA standards ranging from 0.103 to 25 ng/μL (3-fold dilution; six data points) were used to generate the standard curves for the telomere and 36B4 fragments on each 96-well plate. The Tel STD curve was used to measure the telomeric content per sample in kilobases (kb), while the 36B4 STD curve was used to measure the number of diploid genome copies per sample. The qPCR method used to evaluate aTL was adapted in our laboratory, and our results showed reproducible and consistent standard curves for both the telomere and 36B4 (single copy gene) standards (see in Supplementary Information Figures S1 and S2). The cycle threshold (Ct) of the telomere qPCRs ranged from 6 to 15, and all target samples were within the standard linear range. All samples were analyzed in triplicate, with negative and reference controls as well as standard curves. The Ct point of each sample was used to calculate the total aTL in kb per *C. torquatus* diploid genome. Individual samples with a standard deviation of Ct < 1 for the triplicate samples were included in the full analysis.

### Global DNA Methylation Analysis by HPLC

Global DNA methylation (5-mdC) levels were quantified in the isolated DNA of 30 controls (22 females and 8 males) and 35 exposed individuals (30 females and 5 males) based on the proportional quantification of 5-mdC using high performance liquid chromatography (HPLC) as detailed elsewhere^60,61^. Briefly, DNA was hydrolyzed with nuclease P1 and alkaline phosphatase to yield 2′-deoxymononucleosides, which were isolated by HPLC and detected by ultraviolet (UV) light. A mixture of deoxyadenosine, deoxythymidine, deoxyguanosine, deoxycytidine, 5-methyl-2′-deoxycytidine and deoxyuridine (Sigma-Aldrich) was used as a standard. The percentages of global genomic DNA methylation were calculated by the integration of the 5-mdC peak area (obtained from the HPLC analysis) relating to global cytidine (methylated or not). The average for each sample was calculated and duplicated samples, showing a difference in 5-mdC greater than 3% or with low HPLC resolution, were removed.

### Statistical Analysis

The Bartlett-Box test was used to evaluate DNA damage (CA) and the variation in the homogeneity of each site group. The damage index (DI) was calculated based on the number of cells with tails versus those without tails. The statistical evaluation was performed using ANOVA (Dunnet). The Kruskal Wallis test was used to compare significant differences in aTL among the Pelotas, Butiá and Candiota groups. The correlations between telomere length and the other characteristics (sex, age and tissue) were evaluated by Spearman’s test. To statistically analyze the methylation data, we applied nonparametric tests, such as the Mann–Whitney-Wilcoxon test to identify differences between the exposed and control individuals in binary variables or the Kruskal-Wallis test for variables with more than two states. Spearman’s test was also used to examine the correlation of methylation data with tissue, age and sex. Additionally, we evaluated whether there was any correlation between aTL and the methylation data. P ≤ 0.05 was considered statistically significant. Statistical analyses were performed with GraphPad PRISM software, version 5.01 (GraphPad Inc., San Diego, CA).

## Supplementary information

SUPPLEMENTARY INFORMATION - ANTHROPOGENIC EFFECTS ON NATURAL MAMMALIAN POPULATIONS: CORRELATION BETWEEN TELOMERE LENGTH AND COAL EXPOSURE

## Data Availability

The datasets generated during and current study are available from the corresponding author on reasonable request. All data generated or analyzed during this study are included in this published article (and its Supplementary Information files). Readers are welcome to comment on the online version of the paper.
