# Supplementary material for: Anthropogenic Effects on Natural Mammalian Populations: Correlation Between Telomere Length and Coal Exposure
Source: Sci Rep. 2019 Apr 19;9:6325. doi: 10.1038/s41598-019-42804-8 (PMC6474877; doi:10.1038/s41598-019-42804-8)

ANTHROPOGENIC EFFECTS ON NATURAL MAMMALIAN POPULATIONS: CORRELATION BETWEEN TELOMERE LENGTH AND COAL EXPOSURE

Cristina A. Matzenbacher,
Juliana da Silva,
Ana Leticia H. Garcia,
Mónica Cappetta &
Thales R. O. Freitas

SUPPLEMENTARY INFORMATION

The telomere standard curve (Tel STD) was used to measure the telomeric content per sample in kilobases (kb). The cycle threshold (Ct) of the telomere qPCRs ranged from 6 to 15, and all target samples were within the standard linear range. The 36B4 STD curve was used to measure the number of diploid genome copies per sample.

Figure S1. Standard curves used to calculate aTL. The log of kb of telomere standard oligomer DNA.

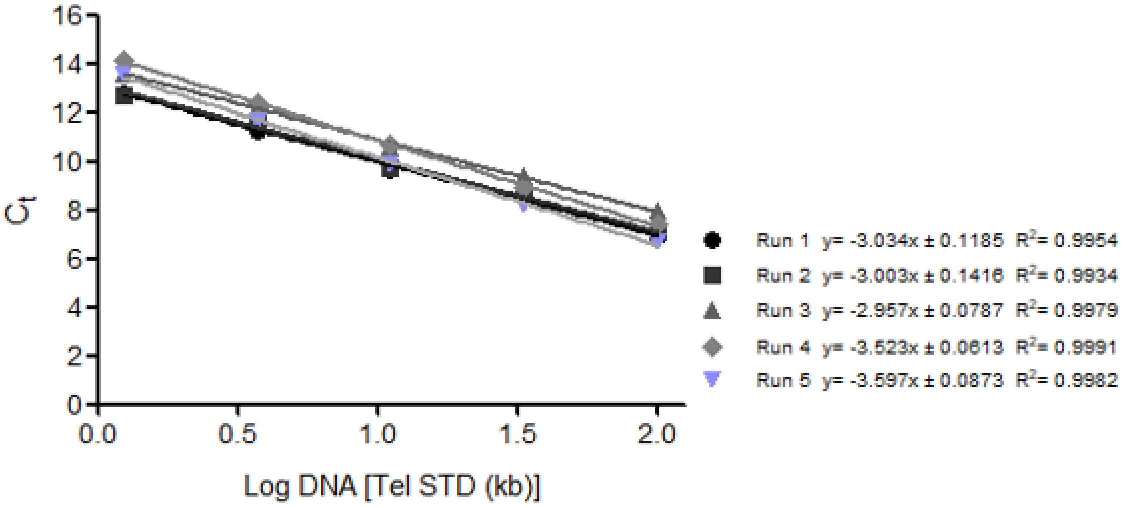

Figure S2. Standard curves used to calculate aTL. The log of kb of 36B4 standard oligomer DNA.

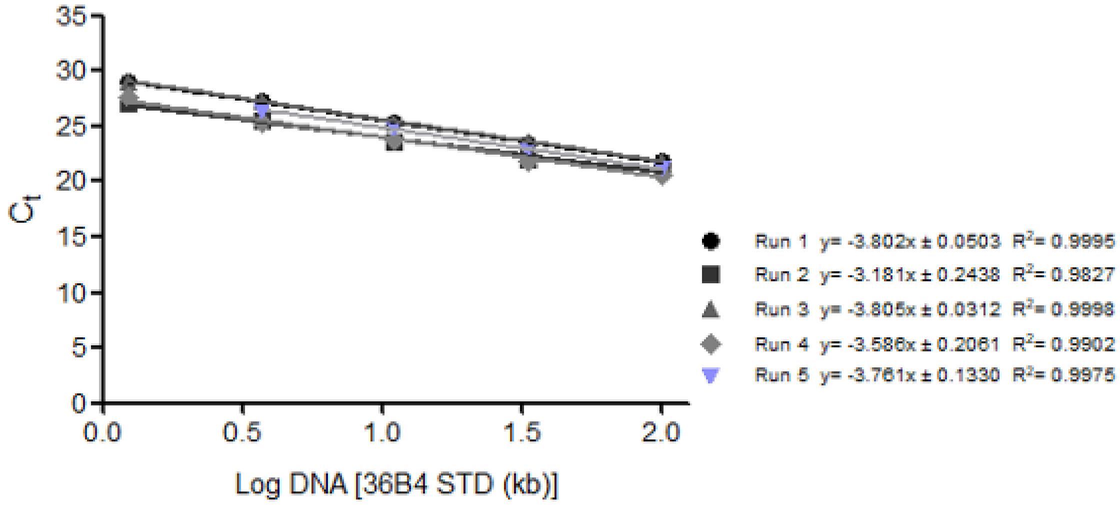

Supplement: Supplementary file 1 — SUPPLEMENTARY INFORMATION - ANTHROPOGENIC EFFECTS ON NATURAL MAMMALIAN POPULATIONS: CORRELATION BETWEEN TELOMERE LENGTH AND COAL EXPOSURE [file 41598_2019_42804_MOESM1_ESM.pdf]
